# Matrix Bound Nanovesicles Modulatory Effect of Inflammation In Vitro in THP-1 Cells

**DOI:** 10.3390/pharmaceutics18060720

**Published:** 2026-06-11

**Authors:** Antonio Giuseppucci, Jianing Chen, George Hussey, Héctor Capella-Monsonís

**Affiliations:** 1 McGowan Institute for Regenerative Medicine, University of Pittsburgh, Pittsburgh, PA 15219, USA; antoniogiuseppucci@gmail.com (A.G.); jianing.chen@duke.edu (J.C.); gsh8@pitt.edu (G.H.); 2 Department of Pathology, University of Pittsburgh, Pittsburgh, PA 15213, USA; 3 Viscus Biologics LLC, Cleveland, OH 44128, USA

**Keywords:** THP-1 cells, macrophages, matrix-bound nanovesicles (MBV), inflammation, vesicle uptake

## Abstract

**Background:** Matrix-bound nanovesicles (MBV) are extracellular vesicles (EVs) that are embedded within the extracellular matrix (ECM), and they have shown immunomodulatory effects in various cell types. The THP-1 cell line is often used to study monocyte and macrophage functions due to its easy culture potential and relatively simple conditioning into different macrophage phenotypes, but the optimal culturing conditions that allow MBV immunomodulation have not been established. **Methods:** In this study, we evaluated different culturing and differentiation conditions of THP-1 cells in which MBVs showed immunomodulatory effects. We also studied the effect of MBVs on relevant inflammation pathways (NF-κB and ERK 1/2). **Results:** Quantification of inflammatory cytokine IL-6 indicated modulation effects by MBVs in the majority of the conditions, but TNF-α showed very limited modulation. ERK1/p44 phosphorylation was significantly increased in MBV groups, but NF-κB protein p65 expression was unaffected. When compared to serum EVs, vesicle uptake by THP-1 cells remained low after 24 h. Multispectral flow cytometry analysis of THP-1 cells exposed to MBV and serum EVs showed internalization of lipids, proteins and RNA within the cells in higher cell proportions, but colocalization of the different vesicle components was not observed. **Conclusions:** Overall, this study provided insights into MBV immunomodulatory effects on THP-1 cells and compared the effects of MBV and serum EVs. Slight differences in modulation were observed between both EV sources, pointing to cargo differences that need further investigation.

## 1. Introduction

Matrix-bound nanovesicles (MBV) are a subset of extracellular vesicles (EVs) that are uniquely embedded within the extracellular matrix (ECM). They range from 20 to 200 nm in size and are known to contain both membrane-embedded and intra-vesicular protein, microRNA and lipid cargo, which are unique to the tissue of origin [1]. Significant work has been done to elucidate the role that MBVs play in the ECM, including their role as an immunomodulatory agent and in the healing process. MBVs have been observed to promote an anti-inflammatory phenotype in myeloid cells, specifically driving a pro-remodeling phenotype in macrophages [2,3], an event required to promote the resolution of inflammation and further functional healing [4]. This effect has been associated with the epigenetic and transcriptomic regulation of immune cells [5,6,7] and has been reported in a wide range of in vivo inflammatory disease state models [6,8,9,10,11]. However, the mechanisms and regulatory pathways by which MBVs cause modulation in myeloid cells is still far from being wholly understood.

THP-1 is an immortalized human leukemia monocytic cell line often used as a model since it mimics morphological and functional features of primary monocytes and macrophages [12]. This cell line is advantageous as a model because of its fast growth and homogeneous genetic background [13]; thus, they are commonly used to study monocyte modulation and macrophage phenotypes [12]. THP-1 cells differentiate into macrophage-like cells upon exposure to phorbol-12-myristate-13-acetate (PMA) and can be further polarized to different macrophage pro-remodeling and pro-inflammatory phenotypes under specific conditions [13]. In fact, THP-1 cells have been employed in previous studies as a model to explore the immunomodulation of ECM materials such as porcine urinary bladder matrix (UBM), where downregulation of inflammation has been reported [14,15,16]. However, many protocols for differentiation of THP-1 cells have been reported in the literature [17,18,19], and these variations, together with variations in culture conditions, impact THP-1 differentiation and derived macrophage behavior. Therefore, taking advantage of THP-1 model plasticity, we ventured to identify the conditions in which MBVs elicit immunomodulation in this cell line.

Specifically, the objective of the present study was to explore the conditions in which MBVs can modulate inflammation in THP-1 cells differentiated with PMA in vitro. In addition, the effect of MBVs on relevant pathways responsible for inflammatory response mediated by toll-like receptors (TLR, i.e., NF-κB and ERK1/2 pathways) were studied, and their uptake and immunomodulatory effects were compared to those observed in EVs derived from serum.

## 2. Material and Methods

### 2.1. Cells and Materials

Unless specified, all materials and reagents were obtained from Sigma-Aldrich, United States. THP-1 cells (ATCC, Manassas, VA, USA) were grown in suspension at 37 °C, 5% CO_2_ in a culturing media containing RPMI 1640 Medium (Gibco, Carlsbad, CA, USA), 10% fetal bovine serum (S11150, Bio-Techne Sales Corp, Minneapolis, MN, USA), 1% Penicillin-Streptomycin (SV30010, HyClone, Logan, UT, USA), and 5 µM of β-mercaptoethanol (M3148, Sigma-Aldrich, Burlington, MA, USA). Media was changed every 2–3 days, and cells were maintained at a maximum density of 1 × 10^6^ cells/mL.

MBVs were isolated from porcine urinary bladder matrix (UBM) as previously described [3,20]. Briefly, UBM obtained from slaughterhouse (6–8 months age pigs) was decellularized by physical delamination, chemical treatment with peracetic acid (Enviroguard MP2, Rochester, NY, USA), and final washes with purified water. Then, the tissue was freeze-dried and micronized using a Wiley mill. The decellularized UBM powder was digested with Liberase^®^ (highly purified Collagenase I and Collagenase II, Roche, Basel, Switzerland) in Tris-HCl buffer at 37 °C overnight with continuous agitation. Digested UBM solution containing the MBVs went through serial centrifugation at 500× *g* for 10 min, 2500× *g* for 20 min, and 10,000× *g* for 30 min. The supernatant was passed through a 0.22-μm filter and then centrifuged at 100,000× *g* (Optima L-90K Ultracentrifuge, Beckman Coulter, Indianapolis, IN, USA) at 4 °C for 70 min to pellet the MBVs. The vesicle pellets were washed and resuspended in phosphate-buffered saline (PBS) and stored at −80 °C for further use. MBV concentration was quantified with nanoparticle tracking analysis using NanoSight NS500 (Malvern Analytical, Westborough, MA, USA).

Serum EVs were isolated from porcine serum (P9783, Sigma-Aldrich, Burlington, MA, USA) following the same centrifugation, ultracentrifugation and filtration protocol described for MBV, and analyzed with nanoparticle tracking. The concentration of protein in both EV populations was quantified using a BCA kit (ThermoFisher Scientific, Waltham, MA, USA).

### 2.2. THP-1 Response to PMA

THP-1 cells were seeded at a density of 1 × 10^6^ cells/well in 48-well plates and incubated in culturing media containing 50 nM, 100 nM, 200 nM, or 320 nM PMA (524400, Sigma-Aldrich, Burlington, MA, USA). Following PMA treatments, adhered cells were rinsed with PBS and were incubated in culturing media for maturation. Six different combinations of PMA treatment time and THP-1 maturation time were tested, including all combinations of PMA treatment time (24 and 48 h) and maturation time after washing (24, 48 and 72 h). For each combination, mature THP-1 cells were incubated in a M1 inflammatory media (serum free THP-1 culturing media containing 100 ng/mL lipopolysaccharide (LPS) and 20 ng/mL interferon-γ (IFNγ)) for 6 h. These inflammatory conditions have been demonstrated to effectively induce an M1-like phenotype in THP-1 cells [16,21]. Non-inflammatory control wells were incubated with serum free THP-1 culturing media. Following polarization, all wells were rinsed with PBS, and half of the M1 wells were further treated with MBVs resuspended in serum free THP-1 media (10^11^ MBV/mL, 250 μL/well) for 24 h. M1 and M0 wells without MBV treatments were maintained in serum free media. Therefore, cells were classified as M0 (non-inflammatory control), M1 (THP-1 cells incubated under inflammatory conditions) and MBV (THP-1 cells incubated under inflammatory conditions and further treated with MBV). Presence of viable adhered cells, morphology and proliferation was monitored during the study to ensure viability of cells. In addition, a preliminary test was performed on the longest time points at three different screening concentrations (i.e., 80, 160 and 320 nM of PMA, Supplementary Figure S1). At the end of the incubation, media was collected from each well and stored at −20 °C for analysis of inflammatory cytokines IL-6 (human) and TNF-α (human), following manufacturer instructions (Fisher Scientific S6050, and R&D Systems STA00D, respectively, from Waltham, MA, and Minneapolis, MN, USA).

### 2.3. MBV Effect on THP-1 Inflammatory Pathways

THP-1 cells were treated with 320 nM PMA for 24 h and matured for 48 h. These conditions were extrapolated from the previous experiment. After inflammatory priming and incubation with MBVs following the procedure described above, inflammatory pathways were assessed with Western blot and inflammatory cytokines.

Western blot analysis was used to evaluate the presence of ERK1 (p44, 9102S), phosphorylated ERK1 (9101S), NF-κB (p-65, 8242S), and phosphorylated NF-κB (3033S). All primary antibodies were purchased from Cell Signaling Technology, Danvers, MA, USA. Briefly, whole protein extracts from THP-1 cells were prepared in RIPA buffer supplemented with phosphatases and protease inhibitors. Protein concentration was determined with a micro BCA kit (Thermo Scientific, Waltham, MA, USA). Then, 5 μg of protein were loaded in precasted SDS–polyacrylamide gel electrophoresis (SDS-PAGE) 4 to 20% gradient gel plate (456-1093, Bio-Rad, Hercules, CA, USA), and SDS-PAGE was performed at 120 V. Later, proteins were transferred to a polyvinylidene difluoride membrane (1620177, Bio-rad, Hercules, CA, USA), and membranes were blocked for 1 h with 5% skimmed milk in tris-buffered saline 0.1% Tween 20 (TBST; Santa Cruz Biotechnology, Dallas, TX, USA) and incubated overnight at 4 °C with 1:1000 dilutions of primary antibodies. Then, membranes were washed three times in TBST and incubated in secondary anti-rabbit or anti-mouse antibody (Dako, Carpinteria, CA, USA) at 1:2000 dilution for 1 h at room temperature. After three further washes in TBST, the signal was developed using an enhanced chemiluminescence substrate (PI34095, Thermo Fisher Scientific, Waltham, MA, USA), and chemiluminescence was measured in the Chemi-Doc measuring system (Bio-Rad, Hercules, CA, USA). After antibody stripping, the procedure was repeated with β-actin (4970T, Cell Signaling Technology, Danvers, MA, USA) primary antibody, and the signal was used to normalize methylated histones readings.

TNF-a and IL-6 production was also quantified for each group using ELISA kits, as per manufacturer instructions (R&D Systems, Minneapolis, MN, USA).

### 2.4. Serum EVs and MBV Inflammation Modulation in THP-1 Cells

THP-1 cells were treated with PMA for differentiations following the same conditions as described previously, and half of the cells were then challenged into an M1-phenotype following the same protocol. Then, M1-challenged and M0 unchallenged cells received one of the following three conditions: serum EVs (10^11^/mL), MBVs (10^11^/mL), or no treatment. For each treatment group and challenge status, half of the cells were treated for 1 h while the other half were treated for 24 h. IL-6 and TNF-α were analyzed as described above.

### 2.5. Uptake of Serum EVs and MBV by THP-1 Cells

Nanovesicles were incubated with carboxyfluorescein diacetate succinimidyl ester (CFSE, C1157, Thermo Fisher Scientific, Waltham, MA, USA) dye for 10 min at 37 °C in aseptic conditions. CFSE-labeled MBV and serum EVs were purified through a Sepharose 2B (Sigma Aldrich, Burlington, MA, USA) size exclusion chromatography (SEC) column and fractions three to five were collected. A control of dye in PBS was also prepared following the same procedure. THP-1 cells were cultured as previously stated. SEC-cleaned, CFSE-labeled MBV or serum EVs, or CFSE dye PBS control were added to cells for 15 min, 30 min, 1 h, or 24 h to both challenged and unchallenged cells. After each respective time point, cells were washed with PBS and treated with Trypsin for 5 min to promote detachment, using the help of repeated pipetting. When detached, cells were analyzed by flow cytometry to quantify the amount of cells that took up MBVs or serum EVs at each time point. Flow cytometry was carried out with a BDFACS Aria II using the software BD FACSDiva 9.0.1 (BD Biosciences, San Jose, CA, USA), and data analysis was performed with FlowJo Software v10.9 (FlowJo LLC, Ashland, OR, USA).

### 2.6. Multispectral Flow Cytometry Imaging in THP-1 Cells Exposed to Serum EVs and MBV

THP-1 cells were cultured, differentiated and challenged (M1 or M0 control) following the same protocol, incubating cells with MBV for 24 h. Previously, MBVs and serum EVs were stained with thiazole, pkh26, and Ghost dye to stain for RNA, lipids, and proteins, respectively, following manufacturer instructions (Thermo Fisher Scientific, Waltham, MA, USA). Then, EVs were washed by SEC as described above. Serum EVs and MBVs stained with single dyes were used for compensation. Cells were then fixed in 1% paraformaldehyde (PFA), centrifuged at 500× *g* and resuspended in 100 µL of PBS at 20 × 10^6^ cells/mL, then transferred to a round-bottom 96-well plate. Cells were analyzed with an ImageStreamX^®^Mk II Flow Cytometer (Cytek, Fremont, CA, USA). Quantification of total intake of each tagged dye, internalization ratio and co-localization ratio were quantified using IDEAS^®^ software v6.2 (Cytek, Fremont, CA, USA).

### 2.7. Statistical Analysis

Statistical analysis was performed with GraphPad Prism 9 (GraphPad Holdings LLC, Boston, MA, USA). Normal distribution of data was assessed using a Shapiro–Wilk test. One-way analysis of variance (ANOVA) and Fisher’s least significant difference post hoc analysis were used to assess statistical significance for normally distributed data, which was assumed when *p* < 0.05. Kruskal–Wallis and Dunn’s post hoc nonparametric tests were used for nonnormally distributed data.

## 3. Results

### 3.1. PMA Concentration and Maturation Condition TNFa and IL6 Release in THP-1 Cells

**Figure 1:**
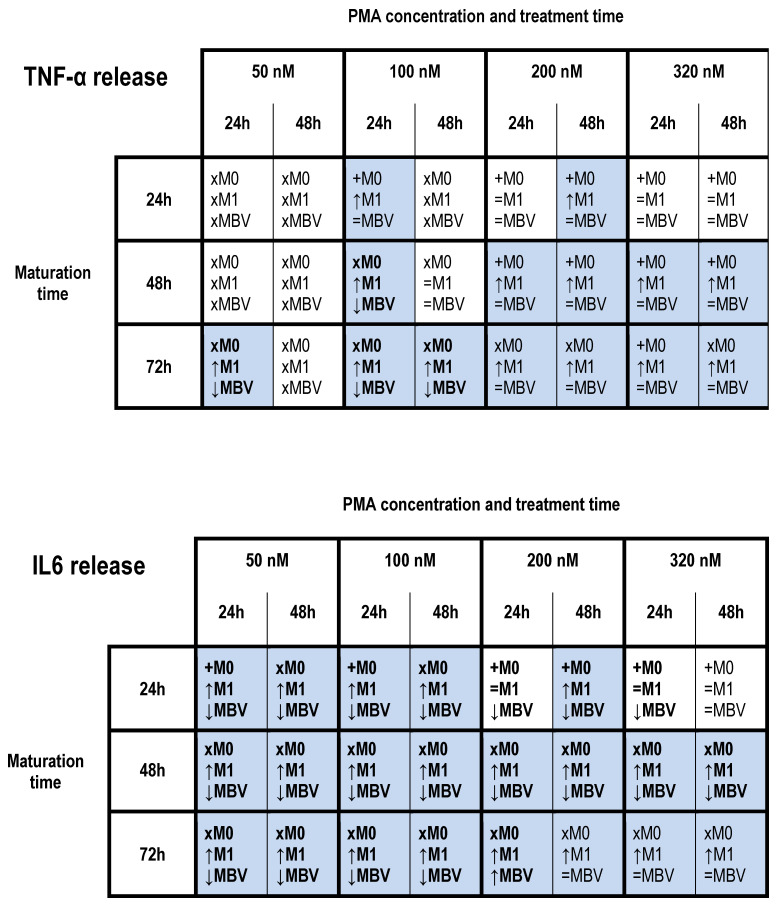
Inflammatory cytokine release in THP-1 cells under different differentiation, maturation and exposure to MBV conditions. Blue: M1 higher cytokine release than M0. Bold: MBV lower cytokine release than M1. X indicates near 0 readings, + indicates a noticeable positive signal for an inflammatory cytokine in M0 condition, ↑ indicates higher production than the condition above (M0 for M1, M1 for MBV), ↓ indicates lower production than the condition above, and = indicates similar production to the condition above. Full graphs and comparisons can be found in Supplementary Figure S2. Indications based on statistical significance (*p* < 0.05, *n* = 4).

The release of key inflammatory cytokines IL-6 and TNF-α by THP-1 cells was assessed under various concentrations of PMA (50, 100, 200 and 320 nM), incubation times (24 and 48 h) and maturation times (24, 48 and 72 h) (Figure 1). This release was measured in naïve THP-1 cells (M0), THP-1 cells exposed to LPS and IFNγ (M1) and M1 THP-1 further treated with MBV (MBV). TNFα release only reached noticeable concentrations, in general lines, in concentrations higher or equal to 100 nM of PMA. Interestingly, high levels of TNFα were also observed in M0 THP-1 cells when exposed to 200 nM and 320 nM PMA. In addition, maturation times of 48 or 72 h were required to observe significant differences in TNFα production between M0 and M1 THP-1 cells. On the other hand, IL-6 release presented a more consistent pattern, with no reading observed in M0 cells and a significant increase in M1 cells observed across most conditions, except for 24 h PMA treatment time followed by 24 h maturation.

Interestingly, MBVs had a very limited effect on TNFα modulation among different conditions, with only a noticeable decrease in production compared to M1 cells in 100 nM PMA conditions, after 48 h maturation. On the other hand, IL-6 modulation by MBVs was evident in most of the conditions, being ineffective only at the highest maturation times and concentrations.

### 3.2. MBV Modulate ERK1/p44 Phosphorylation in PMA-Differentiated THP-1

**Figure 2:**
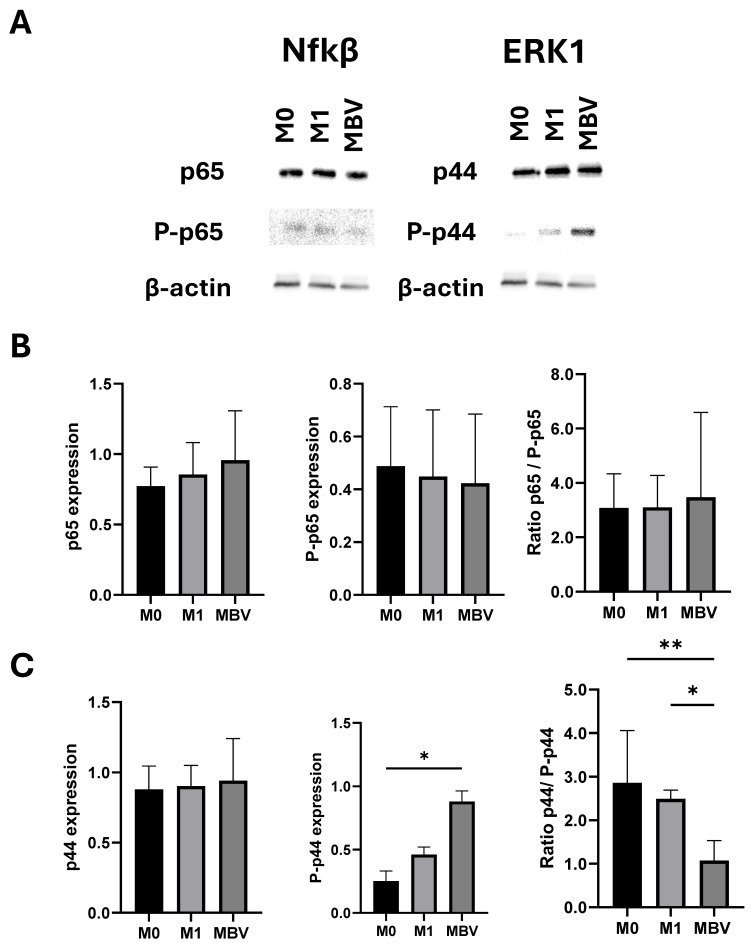
Western analysis of phosphorylation in NF-κB and ERK1 pathways of THP-1 cells under MBV modulation. p65 phosphorylation was unaffected by inflammatory conditions and MBVs, whereas an increase in p44 phosphorylation could be observed when exposed to MBVs and inflammation (**A**). Quantification by densitometry confirmed these observations (**B**,**C**), showing a significant increase in P-p44 and subsequent decrease in p44/P-p44 ratio (**C**). Data are shown as mean ± standard deviation. * and ** indicate *p* < 0.05 and 0.01, respectively (*n* = 3).

In the following experiments, we chose conditions of PMA at 320 nM for 24 h differentiation and 48 h maturation. This condition presented a distinct response between TNF-α (no observed effect of MBVs, while still clear presence of inflammation) and IL-6 (clear effects on regulation by MBV), representing a good candidate for differential responses within inflammatory pathways, which could then be correlated with the release of cytokines TNF-α and IL-6. Western blot analysis (Figure 2A) revealed that phosphorylation of p65, a key protein subunit of NF-κB, remained unaffected in both M1 and MBV conditions, as confirmed by densitometry analysis of p65 and P-p65 (Figure 2B). On the other hand, ERK1/p44 phosphorylation increased in M1 (although not-significantly) and MBV conditions (*p <* 0.05, Figure 2C). This modulation in ERK/p44 phosphorylation by MBVs was further confirmed when comparing the ratio of p44 to its phosphorylated counterpart, which was significantly reduced compared to M0 and M1 conditions (*p <* 0.01 and *p <* 0.05, Figure 2C). In these conditions, IL-6 was found to be downregulated by MBVs, but not TNF-α (Figure 1), which may indicate a modulation in the ERK1 pathway exerted by MBVs. However, it is important to point that phosphorylation of both p44 and p65 are generally linked to an increase in IL-6 and TNFα secretion in TLR-activated inflammatory pathways [22,23,24], which was not observed in this study, most likely because of PMA conditioning.

### 3.3. MBV and Serum EVs Effect on THP-1 Cells Present Small Differences in THP-1 Modulation

**Figure 3:**
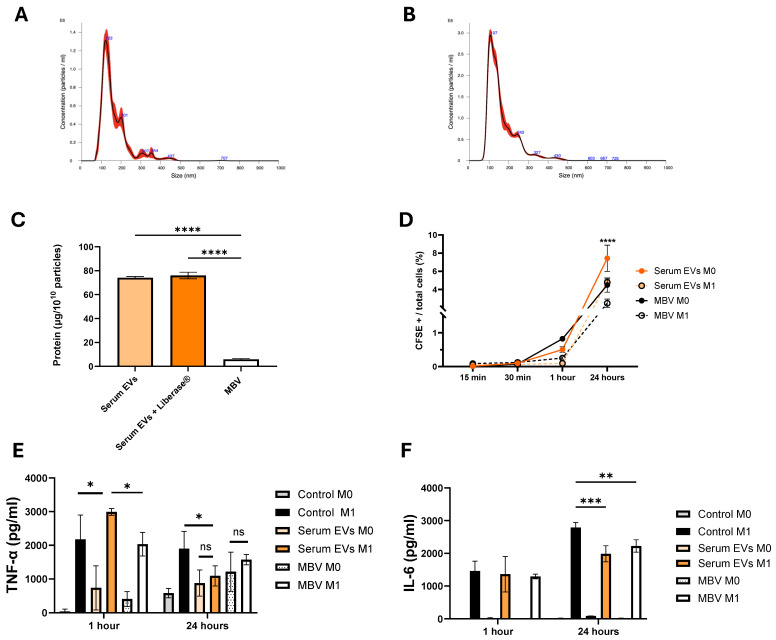
MBV and serum EV characterization, uptake and immunomodulation in THP-1 cells. Both MBVs (**A**) and porcine serum EVs (**B**) showed small EV size distribution, peaking at 100 nm. Protein content analysis showed a higher content in serum EVs (**C**), even after protease treatment of serum EVs. Uptake by THP-1 cells was measured by flow cytometry in both M0 and M1 states after exposure to CFSE-tagged vesicles (**D**). Uptake was below 1% in all groups until 1 h of exposure, and increased after 24 h, being the highest observed in the serum EVs M0 group. TNF-α release (**E**) modulation was observed in the serum EVs group after 1 and 24 h. IL-6 release modulation (**F**) was observed in both serum EVs and MBVs, but only after 24 h of exposure. Data are shown as mean ± standard deviation (*n* = 3). *, **, *** and **** indicate *p* < 0.05, 0.01, 0.001 and 0.0001 respectively, ns indicates “not significant” (*n* = 3).

The protocols for EV isolation used in the present study have been previously reported to yield characteristic and intact EVs for both free-media EVs and MBVs [1,20,25,26], as established by electron microscopy in these reports. Porcine serum-derived EVs were compared to MBVs in size and protein content. Both EV populations presented a similar size distribution as measured by NTA (Figure 3A,B). However, protein per particle content was much higher in serum EVs (Figure 3C, *p <* 0.0001). Since MBVs were isolated by digesting the proteins of the ECM, we hypothesized that this difference could be due to enzyme digestion of proteins surrounding the EVs. However, serum EVs treated with the protease cocktail did not decrease their protein content, indicating that EVs and MBVs present very distinct protein cargo proportions.

We next used flow cytometry to quantify EV and MBV uptake in cells exposed to carboxyfluorescein diacetate succinimidyl ester (CFSE)-tagged MBVs and serum EVs (Figure 3D and Supplementary Figure S3). No differences in quantified uptake were observed after 30 min or 1 h, where the uptake ranged from 0.1 to 0.8% in all groups. However, at 24 h an increase in uptake was observed in all groups, but only to 4–8% percent. After 24 h, THP-1 cells in the M0 state showed a higher uptake than their M1 counterparts, for both MBVs and serum EVs (*p* < 0.0001). The highest uptake was observed in THP-1 M0 cells exposed to serum EVs. TNF-α and IL-6 cytokines release was also assessed in all groups after 1 and 24 h (Figure 3E,F). TNF-α release was unaffected by MBV treatment after 1 and 24 h (Figure 3E), whereas serum EVs slightly increased the release of TNF-α after 1 h compared to M1 control and M1 MBV groups (*p* < 0.05). At 24 h, however, TNF-α levels in the serum EVs M1 group were lower than in the control M1 (*p* < 0.05), showing a modulation of this cytokine and a reduction from the reading at 1 h. Interestingly, no differences (*p* > 0.05) between M0 and M1 were observed in either MBV or serum EV groups. IL-6 release (Figure 3F) only showed modulation after 24 h in both serum EVs (*p* < 0.001) and MBV (*p* < 0.01) groups.

### 3.4. Multispectral Flow Cytometry Imaging

**Figure 4:**
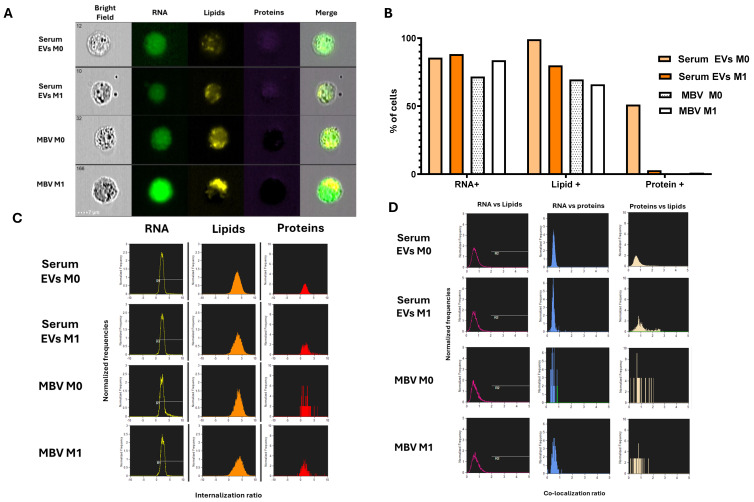
Multispectral flow cytometry imaging of MBVs and serum EVs showed different uptake patterns between vesicle components. MBVs and serum EVs used to treat THP-1 cells were stained with thiazole, pkh26 and Ghost dye to stain for RNA, lipids and proteins, respectively. Representative image of single THP-1 cells (**A**) showing stains for tagged RNA, lipids and proteins in THP-1 cells that have taken up serum EVs, but only RNA and lipids in those that have taken up MBVs. RNA and lipids were observed in more than 70% of cells in all groups (**B**), while proteins were only observed in 50% and <5% in M0 and M1 THP-1 cells respectively. Internalization (**C**) and co-localization (**D**) assessment showed internalization of all EVs components, but no co-localization.

Previous studies have suggested that CFSE-tagged MBVs could be an inaccurate method for measuring uptake in cells by flow cytometry [5], which could be impacting the assumptions and observations in this study. Therefore, we carried out a pilot multispectral flow cytometry analysis in both M0 and M1 THP-1 cells exposed to MBV and serum EVs for 24 h. Both EV types were stained for lipids, proteins and RNA using specific fluorescent dyes (Figure 4).

Representative imaging of each group (Figure 4A) revealed that RNA tagged from vesicles was found within the full cytosol of THP-1 cells, with some concentrated sublocations, whereas lipids derived from both MBVs and serum EVs seemed to concentrate in subcellular locations. Protein staining was only observed in serum EV groups, with no particular pattern of internalization. The proportion of cells positive for each staining and group is summarized in Figure 4B. Interestingly, RNA and lipids were internalized by >70% of THP-1 cells in all groups. However, protein was only internalized by 50% of M0 THP-1 and <5% M1 THP-1 cells when exposed to serum EVs, showing no signal (<1% of cells) in THP-1 cells exposed to MBVs. Images captured were further analyzed to quantify the ratio of internalization of each molecule (Figure 4C) and their co-localization (Figure 4D). All molecules detected showed an internalization coefficient >1, indicating an internalization within the cells rather than staying attached to the cellular membrane. Regarding colocalization of molecules, none of the combinations between lipids, proteins and RNA showed a noticeable population of cells with a coefficient above 2, indicating that colocalization within the cells was not observed. A very limited number of cells showed colocalization in protein and lipids within M1 THP-1 cells exposed to serum EVs (Figure 4D).

## 4. Discussion

MBV-driven immunomodulation was first demonstrated in 2016 [27] when researchers showed how isolated MBVs replicated the immunomodulatory properties of their same-source ECM, demonstrating the critical role of MBVs in the regenerative potential of ECM bioscaffolds [3]. Since then, several studies have reported MBV immunomodulation in in vitro and in vivo studies. However, MBVs are complex in nature while characteristically unique [25], making it challenging to elucidate the mechanisms behind their immunomodulation. In fact, these mechanisms together with those related to MBV uptake are far from being completely understood [28]. Herein, in this study we ventured to assess the effects of MBVs on an established macrophage-like cell model inflammatory pathway, while also comparing their immunomodulation and uptake of serum EVs.

THP-1 cells have been widely used to study inflammatory modulation of different compounds [12,29,30]. However, protocols implementing PMA to differentiate THP-1 in a macrophage-like state broadly vary between studies. In the present study, we showed that PMA concentration and exposure time, together with maturation, influence THP-1 cells’ capacity to respond to LPS, a well-known TLR4 signaling activator. Optimization experiments discarded any sign of cytotoxicity but revealed a lower proliferation in higher concentrations of PMA, probably related to higher levels of differentiation. Interestingly, these differences were not related to lower production of cytokines. The different PMA protocols also influenced the responsiveness of THP-1 cells to MBV modulation in means of IL-6 and TNF-α release. Interestingly, longer maturation times and higher PMA concentrations seem to be required to facilitate THP-1 TNF-α production in the presence of LPS. On the other hand, IL-6 production in inflammatory conditions was observed at low concentrations and short maturation times. PMA acts as an activator for the protein kinase C by mimicking diacylglycerol [31,32], which is also involved in TLR4-driven inflammatory response pathways such as NF-κB. It has previously been shown that PMA differentiation exposure influences TNF-α release and expression in THP-1 cells [33]. Similarly, PMA also influences IL-6 production through both AP-1 and NF-κB transcription factors [34]. Considering how intimately cell cycle and response to inflammation processes are related to NF-κB, MAPK, and AP-1 pathways, it is expected that PMA protocol for THP-1 differentiation influences the response to LPS.

Porcine UBM-derived MBV modulation on TNF-α was observed in very few conditions, mostly related to long maturation times and low PMA concentration. Conversely, IL-6 was modulated by MBVs in most conditions. We further analyzed downstream TLR4 activation in inflammatory pathways under a specific condition (i.e., PMA at 320 nM for 24 h differentiation and 48 h maturation) that showed modulation of IL-6 but no modulation of TNF-α, while M1 polarization was still obvious. Whereas the NF-κB phosphorylation was unaffected by MBVs, their presence was related to an increase in p44 phosphorylation. These results suggest that MBV immunomodulation could be linked to a modulation of the ERK pathway, and consequently the AP-1 transcription factor. p44 phosphorylation is generally related to an increase in inflammatory cytokine production, but MBV exposure after inflammatory conditions increased p44 phosphorylation while consistently decreasing IL-6 production in the present study. Jo et al. [35] demonstrated the crucial role of the ERK pathway in modulating M1 polarization of THP-1 cells, especially after the onset of differentiation with PMA. Moreover, ERK1 phosphorylation has also been reported to be paramount in M2 polarization of THP-1 cells [36], which would relate to the decrease in inflammatory cytokines in the present study. Authors of this study linked ERK signaling in M2 polarization to PPARγ and RA signaling. It could be then hypothesized that different components of MBV have different effects on inflammatory pathways at different levels (i.e., expression, transcription) that elicit a polarization to pro-remodeling phenotypes. In fact, it has recently been reported that MBVs trigger epigenetic modulation in various transcription factor-binding sites of myeloid cells [5], including Jun and Fos (AP-1 pathway) in differentiated macrophages. These findings suggest that future studies investigating MBV immunomodulation in THP-1 cells should study the correlation between ERK, PPARγ, RA and AP-1 during both M1-like and M2-like polarization in the presence of MBVs. Additionally, future studies can investigate these at different PMA conditions, which highly influences differentiation and polarization of THP-1 cells.

We next compared how MBVs and free media EVs from the same source species (porcine) influenced the response to inflammatory conditions in THP-1 cells and how this related to their uptake. Both vesicle populations elicited similar responses regarding IL-6 production, while TNF-α was only modulated by serum EVs, showing differences at early (higher release than control and MBV groups) and later (lower release than control) stages of inflammation. A recent study showed how MBV and free-media EVs from the same cell source had in fact similar but different and complementary immunomodulatory effects [37]. It is likely that differences in composition between free-media EVs and MBVs [1,25] are responsible for these differences, while both sources show immunomodulatory properties. Several studies have reported immunomodulation in THP-1 cells with different EVs. Similarly to serum EVs in the present study, Wang et al. reported that mesenchymal stromal cell-derived EVs upregulated CD206 and CD163 expression and increased anti-inflammatory cytokine IL-10 secretion in M1 THP-1 cells [38]. A reduction in the secretion of pro-inflammatory cytokines TNF-α and IL-1β, and a decrease in NO production were also observed. The work of Yushi et al. [39] also reported TNF-α reduction in M1 THP-1 cells treated with amniotic fluid-derived EVs, while interestingly reporting no effects on IL-6. These slight differences between studies demonstrated that the source of EVs, and therefore their composition, have an impact on the mechanism by which they elicit immunomodulation on THP-1 cells. It also should be considered that the species source could have an effect on the immunomodulatory properties of EVs. A recent report demonstrated tissue-specific molecular signatures in MBVs that could have a link to their cell-modulating properties [1]. Therefore, one could also expect differences in composition between species. Investigating the relevance of these differences between tissue and species on the regenerative potential of free media EVs and MBVs should be matter of future research. Nonetheless, immunomodulation seems to be a common property of EVs unless sourced from pathogens or primed sources [40,41,42]. Noticeably, the present study is limited to only two main inflammatory cytokines (i.e., IL-6 and TNF-α), which is a reductionist approach to studying the modulation of the inflammatory response. However, the study of fewer cytokines allows the inclusion of more conditions, simpler observations, and therefore a general overview of the immunomodulatory effects studied. Following studies should expand the investigation to a wider array of inflammatory and anti-inflammatory cytokines, both at release and expression level, enabling a more comprehensive understanding of the events elicited by MBVs.

Protein content in serum EVs and MBVs was a major difference between EV sources observed in this study. Other studies in the literature have quantified MBVs [1,20] and serum EVs [1,43,44] from other sources in comparable ranges, where the variability in results is noticeable. However, no major differences in uptake reading were observed by CFSE-tagged flow cytometry, which would be unexpected given CFSE’s need for permeation, activation by esterases and coupling to amine groups (i.e., proteins) to generate a signal. However, CFSE may not be a suitable dye for certain EVs and in particular MBVs, as a recent study suggested [5]. In fact, protein labeling in multispectral flow cytometry imaging in the present study showed a different pattern of intake to that observed with CFSE only. Interestingly, very low intake of protein could be observed for MBV groups, as opposed to lipid and RNA, which ranged from 70 to 90% uptake, similar to that observed in another study involving THP-1 cells and EVs [39]. In addition, it could be observed that THP-1 cell machinery did uptake the different EVs and sort their components in different subcellular compartments, since no colocalization was observed in any of the groups. Further studies combining specific uptake inhibitors and multispectral flow cytometry imaging could help understanding the functioning of such mechanisms.

## 5. Conclusions

The current study described for the first time the immunomodulation of MBVs on the model THP-1 cell line, while highlighting the importance of the differentiation protocol in the model. In addition, these effects, and the uptake, were compared to those of serum EVs, with slight differences observed. Future studies must aim to fill the gaps in molecular pathways related to TLR4 inflammation that the present report has identified. Using specific phosphorylation inhibitors in different protocols of differentiation could be an interesting approach to doing so. In addition, the use of multiplex ELISA for inflammatory and anti-inflammatory cytokines would add valuable information to the mechanism of action of MBVs. Finally, the present study demonstrates that the THP-1 model, while useful, presents an evident sensitivity to protocol conditions in terms of incubation times and concentrations; therefore, results of the present study must be interpreted carefully when comparing them to other studies.

## Data Availability

All the data to reproduce the manuscript is included in the main text or Supplementary Materials. Additional information can be available upon request.

## Supplementary Materials

The following supporting information can be downloaded at: https://www.mdpi.com/article/10.3390/pharmaceutics18060720/s1, Supplementary Figure S1. Preliminary screening of THP-1 showed no cytotoxic events elicited by PMA nor UBM MBVs, but a reduction in the amount of cells present under high PMA conditions. All concentrations of PMA were tested for 48 h incubation with PMA, and 72 h maturation. All conditions showed viable adhered cells after incubation and washing with PBS. For DNA content analysis, water was added to promote osmotic lysis, and cells were completely lysed using a sonicator. Then, samples were used to measure DNA content with PicoGreen^®^ Assay (ThermoFisher Scientific, USA), correlating to proliferation/presence of viable cells (*n* = 4). Supplementary Figure S2. ELISA of TNF-**α** and IL-6 released by THP-1 cells under different differentiation, maturation and exposure to MBV conditions. TNF-α concentrations released by THP-1 cells after 24 h or 48 h of PMA treatment followed by 24 h, 48 h, or 72 h of maturation (A–F). IL-6 concentrations released by THP-1 cells after 24 h or 48 h of PMA treatment followed by 24 h, 48 h, or 72 h of maturation (G–L). Data shown as mean ± standard error of the mean (SEM), *n* = 4. Supplementary Figure S3. Histogram showing FITC-A intensity profile and counts after flow cytometry of serum EVs treated M0 THP-1 cells (A), MBV-treated M0 THP-1 cells (B), serum EVs treated M1 THP-1 cells (C), and MBV-treated M1 THP-1 cells (D).
