# Supplementary material for: Matrix Bound Nanovesicles Modulatory Effect of Inflammation In Vitro in THP-1 Cells
Source: Pharmaceutics. 2026 Jun 11;18(6):720. doi: 10.3390/pharmaceutics18060720 (PMC13306160; doi:10.3390/pharmaceutics18060720)
Supplement: Supplementary file 1 [file pharmaceutics-18-00720-s001.zip › pharmaceutics-4260842-supplementary.pdf]

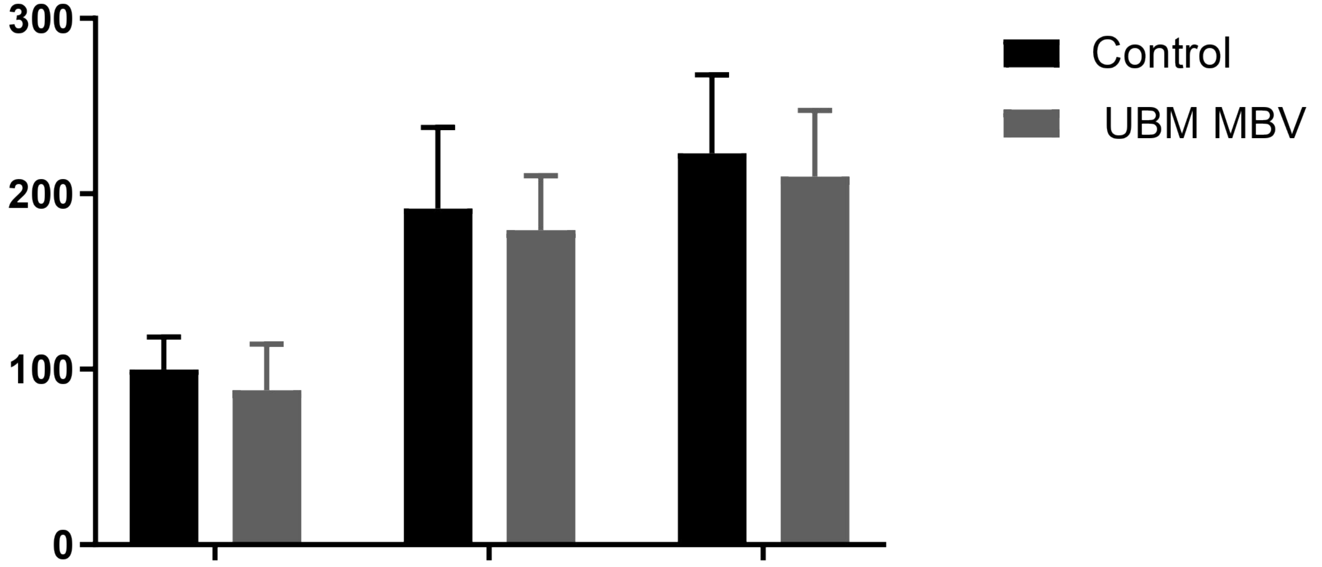

Figure S1

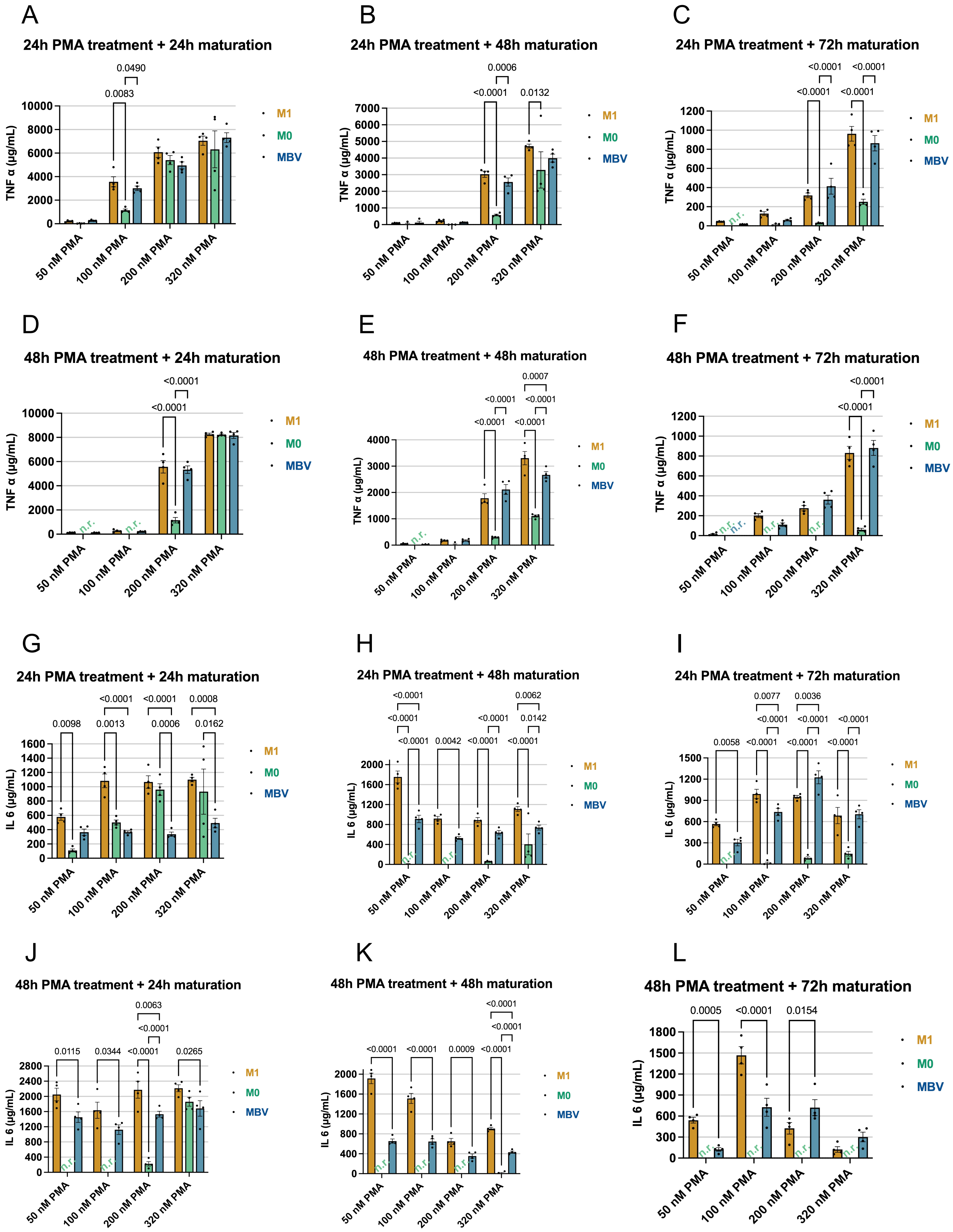

PMA concentration and treatment time

TNF-α release (μg/mL)		50 nM		100 nM		200 nM		320 nM	
		24h	48h	24h	48h	24h	48h	24h	48h
Maturation time 24h	M1	212.8	127.6	3562.1	269.5	6076.9	5569.9	7050.1	8255.2
	M0	26.8	n.r.	1149.2	n.r.	5407.6	1155.2	6318.4	8208.6
	MBV	270.9	113.9	3010.8	211.2	4958.2	5336.1	7313.3	8162.1
48h	M1	91.7	54.4	**225.7**	175.7	3030.3	1785.1	4718.1	3306.6
	M0	35.1	n.r.	**1.7**	5.4	580.3	292.8	3285.5	1103.6
	MBV	128.0	21.7	**126.1**	183.2	2560.9	2115.3	3994.8	2669.4
72h	M1	**44.6**	14.7	**128.4**	**201.1**	318.9	276.3	962.6	831.9
	M0	**n.r.**	n.r.	**9.6**	**n.r.**	28.6	n.r.	251.6	59.7
	MBV	**16.5**	n.r.	**60.7**	**110.2**	414.2	361.9	864.3	881.4

PMA concentration and treatment time

IL-6 release (μg/mL)		50 nM		100 nM		200 nM		320 nM	
		24h	48h	24h	48h	24h	48h	24h	48h
Maturation time 24h	M1	**577.7**	**2049.7**	**1082.8**	**1634.7**	**1067.1**	**2174.3**	**1100.7**	2215.1
	M0	**108.8**	**n.r.**	**500.9**	**n.r.**	**961.2**	**224.1**	**932.1**	1858.2
	MBV	**364.0**	**1452.2**	366.3	**1124.2**	**336.2**	**1531.7**	**493.1**	1683.3
48h	M1	**1752.4**	**1915.5**	**917.2**	**1512.4**	**887.1**	**650.8**	**1109.0**	**904.8**
	M0	**n.r.**	**n.r.**	**n.r.**	**n.r.**	**58.5**	**n.r.**	**402.6**	**8.9**
	MBV	**911.8**	**655.4**	**530.1**	**647.0**	**636.3**	**354.3**	**737.8**	**432.2**
72h	M1	**566.5**	**541.5**	**990.8**	**1468.0**	**949.5**	425.2	685.3	123.4
	M0	**n.r.**	**n.r.**	**10**	**n.r.**	**84.6**	n.r.	147.0	n.r.
	MBV	**303.9**	**124.1**	**736.7**	**726.2**	**1225.9**	720.2	704.5	301.1

Figure S2

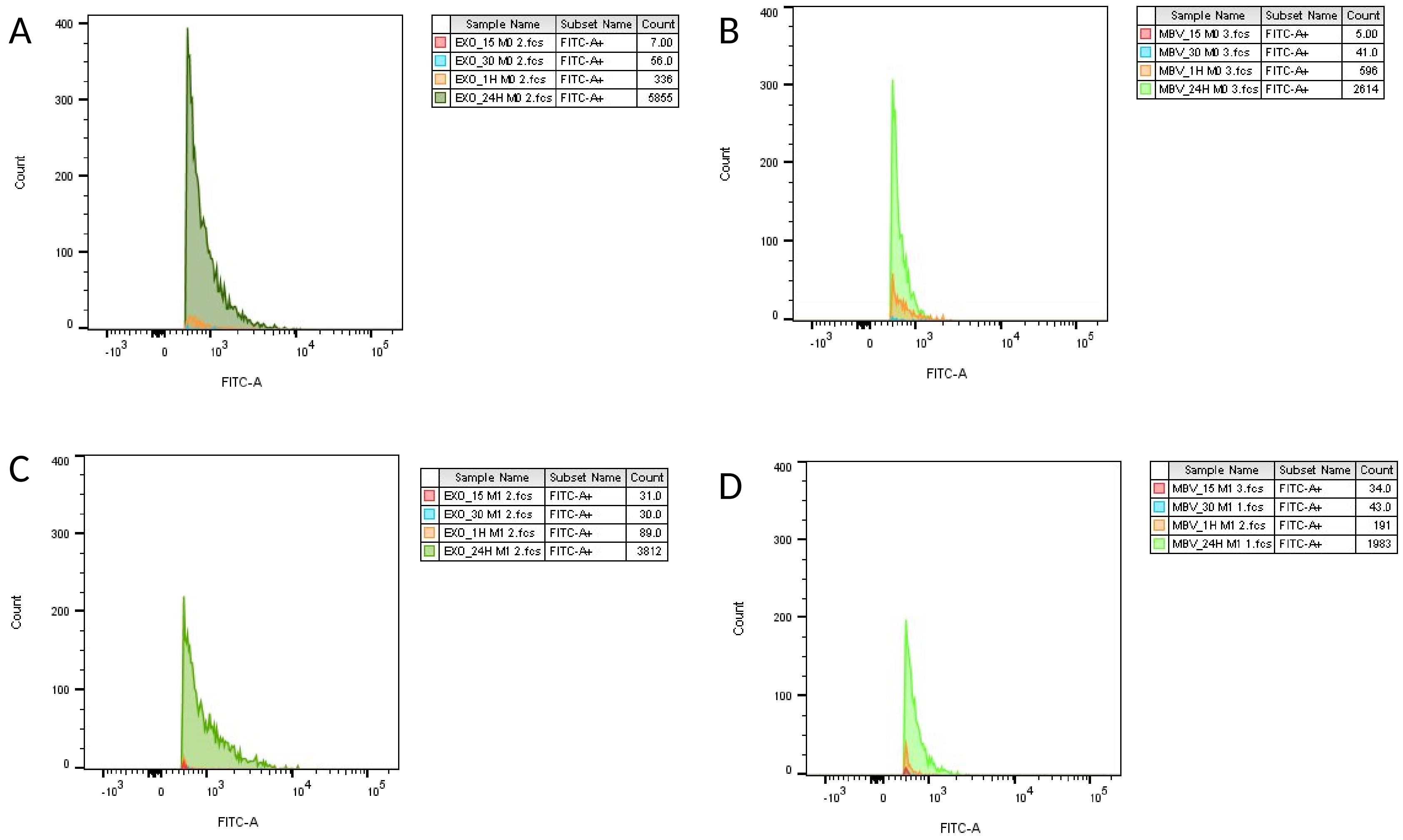
A
Sample Name | Subset Name | Count
EXO_15 M0 2.fcs | FITC-A+ | 7.00
EXO_30 M0 2.fcs | FITC-A+ | 56.0
EXO_1H M0 2.fcs | FITC-A+ | 336
EXO_24H M0 2.fcs | FITC-A+ | 5855
B
Sample Name | Subset Name | Count
MBV_15 M0 3.fcs | FITC-A+ | 5.00
MBV_30 M0 3.fcs | FITC-A+ | 41.0
MBV_1H M0 3.fcs | FITC-A+ | 596
MBV_24H M0 3.fcs | FITC-A+ | 2614
C
Sample Name | Subset Name | Count
EXO_15 M1 2.fcs | FITC-A+ | 31.0
EXO_30 M1 2.fcs | FITC-A+ | 30.0
EXO_1H M1 2.fcs | FITC-A+ | 89.0
EXO_24H M1 2.fcs | FITC-A+ | 3812
D
Sample Name | Subset Name | Count
MBV_15 M1 3.fcs | FITC-A+ | 34.0
MBV_30 M1 1.fcs | FITC-A+ | 43.0
MBV_1H M1 2.fcs | FITC-A+ | 191
MBV_24H M1 1.fcs | FITC-A+ | 1983
Count
FITC-A

Figure S3
